# Supplementary material for: Hematological and CD4+ T- cell count reference interval for pregnant women attending antenatal care at Hawassa University Comprehensive Specialized Hospital, Hawassa Southern Ethiopia
Source: PLoS One. 2021 Apr 8;16(4):e0249185. doi: 10.1371/journal.pone.0249185 (PMC8031454; doi:10.1371/journal.pone.0249185)
Supplement: Attachment — Submitted filename: In-house Editor Response.docx [file pone.0249185.s001.docx]

| **Date:** | Dec 31 2020 04:42AM |
| --- | --- |
| **To:** | "Demissie Assegu Fenta" demissieasegu@yahoo.com |
| **From:** | "PLOS ONE" plosone@plos.org |
| **Subject:** | PLOS ONE: Your submission PONE-D-20-31793R1 |

=============================================
IMPORTANT: PLEASE DO NOT REPLY TO THIS EMAIL
If you are unable to complete any points that are requested in this email, please explain why in the "Enter Comments" tab of the online submission form prior to re-submitting your manuscript. This will enable us to promptly assess your response and progress your manuscript to an Academic Editor at the earliest opportunity.
=============================================

**COVER LETTER**

DEAR: **JOURNAL OF PLOSE ONE EDITORIAL OFFICE.**

We are very grateful to in-house editor who have critically revised the manuscript and provided and raised very important points about the data availability issue and PLOS ONE journal policy which we believe would improve the quality of the manuscript makes us to revise the journal policy in details. Therefore, we have incorporated each and every comment of the Editor in the revised manuscript with highlighting with yellow color. Moreover, point-by-point responses are given below to the raised queries of the editor. We hope that each and every point raised by the Editor is now addressed to the satisfaction of the Editor.

We look forward to hearing from you soon.

Kind Regards

PONE-D-20-31793R1
Hematological and CD4+ T- cell count reference interval for pregnant women attending antenatal care at Hawassa University Comprehensive Specialized Hospital, Hawassa Southern Ethiopia.
Mr Demissie Assegu Fenta

Dear Mr Fenta,

Thank you for submitting your manuscript entitled "Hematological and CD4+ T- cell count reference interval for pregnant women attending antenatal care at Hawassa University Comprehensive Specialized Hospital, Hawassa Southern Ethiopia." to PLOS ONE. Your manuscript files have been checked in-house but before we can proceed we need you to address the following issues:

(1) Thank you for your response regarding our data availability queries. Could you please confirm whether the data provided in the manuscript meet our Minimal Data Set requirement, or if additional restricted data are necessary for replication?

- **Author response:** The comment is well taken and accept he concerns of the in-house Editor.
- **Author action: Yes, we** believe the data provided in the manuscript can meet the minimal data set requirement of the journal, No additional restricted data are available in the corresponding author data set or the university repository for other replication. That is why we give you a full address of the University office address and responsible individuals for conformation.

We define the minimal data set as that which is used to reach the conclusions drawn in the manuscript with related metadata and methods, and any additional data required to replicate the reported study findings in their entirety. This may include:
- The values behind the means, standard deviations and other measures reported

- **Author response:** The comment is well taken and accept he concerns of the in-house Editor.
- **Author action: We also tried to describe the mean values, standard deviation, median values and the like on Table 3 of this manuscript and there is no left over values for the replication of the data.**

- The values used to build graphs

- **Author response:** The comment is well taken we also appreciate your effort on the prevention of data from replication.
- **Author action:** But as you clearly described in the manuscript we haven’t and data left to build graphs for further replication and you can confirm by the given address of the responsible bodies given in the previous response.

- The points extracted from images for analysis

- **Author response:** The comment is well taken we also appreciate your effort on the prevention of data from replication.
- **Author action:** But as you clearly described in the manuscript we haven’t and data left to build graphs for further replication and you can confirm by the given address of the responsible bodies given in the previous response.

If the data currently in your submission constitute your Minimal Data Set, please update your Data Availability Statement to read: "All relevant data are within the manuscript and its Supporting Information files."

- **Author response:** The comment is well taken.
- **Author action:** We tried to update our data availability statement in our cover letter and in additional information part as given by the comment. ‘All relevant data are within the manuscript and its Supporting Information files."

If additional restricted data are necessary to meet the Minimal Data Set requirement, please add the following data access information to your Data Availability Statement:
a) The name of the policy, ethics committee, Institutional Review Board, or third-party organization that is imposing sharing restrictions on your de-identified minimal data set.

- **Author response:** The comment is well taken we also appreciate your effort on the prevention of data from replication.

**Author action:** We tried to address about the availability of data in the manuscript for the current study for further clarity we also tried to give confirmatory bodies during the first revision on the editors comment section as Jimma University institute of health and medicine, Vice President for research and community service webpage or (Tel. +251-471112453, Fax: +251-47111) and all data are included in the manuscript and the research was ethically approved by institutional review board of institute of health, Jimma University, Ethiopia with Ref.No.IHRPGD/537/18 and chairperson of Dr. Zeleke Mekonnen (Email: Zeleke.mekonnen@ju.edu.et, Tel:+251471120945).

b) The grounds for restriction (e.g. contains sensitive identifying information).

- **Author response:** The comment is well taken we also appreciate your concern.
- **Author action:** As we tried to mention in the ethical assurance part this work was conducted after ethically approved by the university Institutional review board, there was no grounds for data restrictions except future planed issues for further study.
  c) A non-author point of contact that is able to field data access queries. PLOS ONE's data policy requires a non-author institutional contact for data access in the interest of maintaining long-term data accessibility.
- **Author response:** The comment is well taken.
- **Author action:** we tried to read and address PLOS ONE’s data policy guide and give address of the non –author institutional contact in the comment given above.
  d) Any relevant data set names, variables, accession codes, URLs, DOIs, etc. that an independent researcher would need in order to request your minimal data set.
  **Author response:** The comment is well taken again.

**Author action:** All relevant data is submitted as manuscript in PLOS ONE Journal, there is any data set names, variables, accession codes, URLs, DOI, and etc. for this manuscript.

Once you've provided the necessary information and/or files, we'll be able to proceed with the review process.

Your manuscript has been returned to your account. Please log on to PLOS Editorial Manager at https://www.editorialmanager.com/pone/ to access your manuscript.

Your manuscript can be found in the "Revisions Sent Back to the Author" link under the New Submissions menu. After you have made the changes requested above, please be sure to view and approve the revised PDF after rebuilding the PDF to complete the resubmission process.

Please note that these changes have been requested to comply with submission guidelines and your manuscript will *not* be sent to review until you have fully adhered to our requests. Once your paper has been seen by an Editor we may return it to you for further information or amendments.

We ask that you address this request within 28 days. If you require additional time, please email the journal office. We are happy to grant extensions of up to one month past this due date. If we have not heard from you within 28 days, your manuscript will be withdrawn from Editorial Manager.

Thank you for submitting your work to PLOS ONE.

Kind regards,

Orsolya Voros
PLOS ONE

__________________________________________________
In compliance with data protection regulations, you may request that we remove your personal registration details at any time. (Use the following URL: https://www.editorialmanager.com/pone/login.asp?a=r). Please contact the publication office if you have any questions.
