# Supplementary material for: Hematological and CD4+ T- cell count reference interval for pregnant women attending antenatal care at Hawassa University Comprehensive Specialized Hospital, Hawassa Southern Ethiopia
Source: PLoS One. 2021 Apr 8;16(4):e0249185. doi: 10.1371/journal.pone.0249185 (PMC8031454; doi:10.1371/journal.pone.0249185)
Supplement: Attachment — Submitted filename: Response to Reviewers.docx [file pone.0249185.s002.docx]

PONE-D-20-31793R1
Hematological and CD4+ T- cell count reference interval for pregnant women attending antenatal care at Hawassa University Comprehensive Specialized Hospital, Hawassa Southern Ethiopia.
PLOS ONE

Dear Dr. Fenta,

Thank you for submitting your manuscript to PLOS ONE. After careful consideration, we feel that it has merit but does not fully meet PLOS ONE’s publication criteria as it currently stands. Therefore, we invite you to submit a revised version of the manuscript that addresses the points raised during the review process.

Please submit your revised manuscript by Mar 15 2021 11:59PM. If you will need more time than this to complete your revisions, please reply to this message or contact the journal office at plosone@plos.org. When you're ready to submit your revision, log on to https://www.editorialmanager.com/pone/ and select the 'Submissions Needing Revision' folder to locate your manuscript file.

Please include the following items when submitting your revised manuscript:

- A rebuttal letter that responds to each point raised by the academic editor and reviewer(s). You should upload this letter as a separate file labeled 'Response to Reviewers'.
- A marked-up copy of your manuscript that highlights changes made to the original version. You should upload this as a separate file labeled 'Revised Manuscript with Track Changes'.
- An unmarked version of your revised paper without tracked changes. You should upload this as a separate file labeled 'Manuscript'.

If you would like to make changes to your financial disclosure, please include your updated statement in your cover letter. Guidelines for resubmitting your figure files are available below the reviewer comments at the end of this letter.

If applicable, we recommend that you deposit your laboratory protocols in protocols.io to enhance the reproducibility of your results. Protocols.io assigns your protocol its own identifier (DOI) so that it can be cited independently in the future. For instructions see: <http://journals.plos.org/plosone/s/submission-guidelines#loc-laboratory-protocols>

We look forward to receiving your revised manuscript.

Kind regards,

Frank T. Spradley
Academic Editor
PLOS ONE

**REBUTAL LETTER**

Dear: **Journal of Plos One Editors and reviewers.**

We are very grateful to the editors and reviewers who have critically revised the manuscript and provided and raised very important points, which we also believe would improve the quality of the manuscript. . Therefore, we have incorporated each and every comment of the Editor in the revised manuscript with highlighting with yellow color. Moreover, point-by-point responses are given below to the raised queries of the editor. We hope that each and every point raised by the Editor and the reviewers will now addressed to the satisfaction of the quires. .

We look forward to hearing from you soon.

Kind Regards

Reviewers' comments:

Reviewer's Responses to Questions

**Comments to the Author**

1. If the authors have adequately addressed your comments raised in a previous round of review and you feel that this manuscript is now acceptable for publication, you may indicate that here to bypass the “Comments to the Author” section, enter your conflict of interest statement in the “Confidential to Editor” section, and submit your "Accept" recommendation.

Reviewer #1: (No Response)

**Response: Thank you for your engorgement,** we tried to submit the conflict of interest statement in supporting information document in the previous review as the authors have declared that no competing interests exist”.

2. Is the manuscript technically sound, and do the data support the conclusions?

The manuscript must describe a technically sound piece of scientific research with data that supports the conclusions. Experiments must have been conducted rigorously, with appropriate controls, replication, and sample sizes. The conclusions must be drawn appropriately based on the data presented.

Reviewer #1: Yes

Response: The comment is well taken and thank you for your constructive comment

3. Has the statistical analysis been performed appropriately and rigorously?

Reviewer #1: Yes

  Response: The comment is well taken and thank you for your constructive comment.

4. Have the authors made all data underlying the findings in their manuscript fully available?

The [PLOS Data policy](http://www.plosone.org/static/policies.action#sharing) requires authors to make all data underlying the findings described in their manuscript fully available without restriction, with rare exception (please refer to the Data Availability Statement in the manuscript PDF file). The data should be provided as part of the manuscript or its supporting information, or deposited to a public repository. For example, in addition to summary statistics, the data points behind means, medians and variance measures should be available. If there are restrictions on publicly sharing data—e.g. participant privacy or use of data from a third party—those must be specified.

Reviewer #1: Yes

  Response: The comment is well taken and thank you for your constructive comment.

5. Is the manuscript presented in an intelligible fashion and written in Standard English?

PLOS ONE does not copyedit accepted manuscripts, so the language in submitted articles must be clear, correct, and unambiguous. Any typographical or grammatical errors should be corrected at revision, so please note any specific errors here.

Reviewer #1: No

Response: We agree with the reviewer’s comment and tried to correct English grammatical issue, and punctuation or spelling, by inviting local senior researchers and language experts as much as possible throughout the manuscript again.

6. Review Comments to the Author

Please use the space provided to explain your answers to the questions above. You may also include additional comments for the author, including concerns about dual publication, research ethics, or publication ethics. (Please upload your review as an attachment if it exceeds 20,000 characters)

Reviewer #1: Thank you for your resubmission and all the effort that was directed towards addressing previous reviewer comments. The results are now much easier to interpret.

Comments

1. To address lingering grammatical errors and further enhance the readability of the manuscript, I would recommend using a language editing service.

Response: We agree with the reviewer’s comment and tried to correct English grammatical issue, and punctuation or spelling, by inviting local senior researchers and language experts as much as possible throughout the manuscript again.

2. There are several sections where the text is highly similar to that of the paper being cited, and the original citations are overlooked. For example, in the Introduction the sentence "Pregnancy is often described as a state of regulated inflammation and is consistently marked by a rise in the total number of circulating leukocytes, primarily due to the expansion of neutrophils and monocytes (14)" is identical to the source paper and does not include the original citations for each of these statements. This issue should be addressed throughout the paper.

Response: We agree with the reviewer’s comment and concern tried to rephrase again the indicated sentence based on the comment given.

7. PLOS authors have the option to publish the peer review history of their article ([what does this mean?](https://journals.plos.org/plosone/s/editorial-and-peer-review-process#loc-peer-review-history)). If published, this will include your full peer review and any attached files.

If you choose “no”, your identity will remain anonymous but your review may still be made public.

**Do you want your identity to be public for this peer review?** For information about this choice, including consent withdrawal, please see our [Privacy Policy](https://www.plos.org/privacy-policy).

 Reviewer #1: Yes

PLOS can use my personal information to customize my experience on the PLOS Sites and to facilitate our interactions. Plos can use our personal information for the purpose of processing, reviewing, communicating about, and peer review, for publishing, Plos can also use our personal information as potential or actual role as or reviewer.

[NOTE: If reviewer comments were submitted as an attachment file, they will be attached to this email and accessible via the submission site. Please log into your account, locate the manuscript record, and check for the action link "View Attachments". If this link does not appear, there are no attachment files.]

While revising your submission, please upload your figure files to the Preflight Analysis and Conversion Engine (PACE) digital diagnostic tool, <https://pacev2.apexcovantage.com/>. PACE helps ensure that figures meet PLOS requirements. To use PACE, you must first register as a user. Registration is free. Then, login and navigate to the UPLOAD tab, where you will find detailed instructions on how to use the tool. If you encounter any issues or have any questions when using PACE, please email PLOS at [figures@plos.org](mailto:figures@plos.org). Please note that Supporting Information files do not need this step.
